# HJ-006: A Non-Hallucinogenic Synaptogenic Compound with Rapid and Sustained Antidepressant Efficacy

**DOI:** 10.1192/j.eurpsy.2026.10580

**Published:** 2026-07-31

**Authors:** L. Zeng, Y. Chen, Y. Liu, Y. Geng

**Affiliations:** Jing Medicine, Shanghai, China

## Abstract

**Introduction:**

Current antidepressants (SSRIs/SNRIs) face limitations including delayed onset (4–6 weeks) and modest efficacy (~50% response rates), while serotonergic psychedelics show rapid antidepressant potential via 5-HT2A agonism and synaptic plasticity but are hindered by hallucinogenic activity and 5-HT2B-mediated cardiotoxicity.

**Objectives:**

To address these challenges, we characterize HJ-006, a novel synaptogenic agent, designed to promote neuroplasticity without hallucinogenic or safety liabilities, and antidepressant efficacy.

**Methods:**

Efficacy was evaluated with head twitch response (HTR) in mice (predictive of hallucinogenic activity), and established in vivo models (Forced Swim Test [FST], Tail Suspension Test [TST], Sucrose Preference Test [SPT] in Chronic Unpredictable Mild Stress [CUMS] and corticosterone-induced depression). Safety was evaluated via Conditioned Place Preference (CPP), neurite outgrowth/synaptogenesis assays, multielectrode array (MEA) recordings in CORT-damaged hippocampal neurons, cardiovascular monitoring (non-human primate ECG), and pharmacokinetic profiling.

**Results:**

HJ-006 is an orally active, brain penetrant, small molecule. In vitro, HJ-006 showed significant effects on neurite-outgrowth and synaptogenesis in primary cultures of rat cortical neurons. It demonstrated rapid (30 min post-single dose) FST antidepressant effects in corticosterone-induced depression and reversed CUMS-induced deficits in FST, TST, and SPT, with efficacy sustained ≥8 days, with no hallucinogenic behavior (HTR-negative). Safety profiles included no CPP rewarding effects, enhanced dendritogenesis/synaptogenesis in cortical neurons, rescued CORT-impaired hippocampal activity (30 nM, 15 min post-treatment), 5-HT2B antagonist activity (Ki = 2.18 nM, IC50 = 272.8 nM), favorable brain penetration, CYP3A4 metabolism, no cardiovascular signals in primates, and a 40× safety margin in 14-day toxicity studies.

**Conclusions:**

HJ-006 is a first-in-class non-hallucinogenic neuroplastogen with rapid, sustained antidepressant-like efficacy and improved safety, supporting its potential as a transformative depression treatment.

**Disclosure of Interest:**

None Declared

